# Operationalizing age-friendliness in urban China: a multi-case study of gated retirement and open multi-generational communities

**DOI:** 10.3389/fpubh.2025.1618534

**Published:** 2025-09-24

**Authors:** Yiting Tan, Jianyuan Huang

**Affiliations:** ^1^School of Public Administration, Hohai University, Nanjing, China; ^2^Population Research Institute, Hohai University, Nanjing, China

**Keywords:** age-friendly community, supportive environments, gated retirement community, open multi-generational community, multi-case study

## Abstract

**Background:**

Age-Friendly Communities (AFCs) play a pivotal role in creating supportive social and physical environments, which enable older adults to maintain mobility, independence, healthier living, and successful aging-in-place.

**Methods:**

This study employed a multi-case study method to analyze six comunities and two age-friendly community models in China: Gated Retirement Communities (GRCs) and Open Multi-Generational Communities (OMGCs), which have exhibited different effects in practice. An analytical framework incorporating policies, facilities, services, intergenerational relationships, and sustainability has been established to systematically compare these models, with the aim of identifying some more effective age-friendly measures at the community level.

**Results:**

The research results revealed that GRCs were prone to spatial inequality, idle waste of resources, violation of service commitment, intergenerational exclusion and unsustainability. On the contrary, OMGCs demonstrated better age-friendliness and stronger vitality.

**Conclusion:**

OMGCs are more supportive and age-friendly than GRCs. Some key priorities and effective measures for the development of AFCs have been obtained from these communities, offering valuable insights for Asian nations and developing countries seeking to advance age-friendly initiatives.

## 1 Introduction

In 2005, the World Health Organization (WHO) launched its age-friendly initiative, establishing comprehensive guidelines that encompass eight domains of livability (e.g., built environment, social infrastructure, service systems). The explicit objective was to create a social and physical environment that is more supportive and responsive, inherently embodying fairness and diversity, thus improving health and well-being among older adults (1–3). As the movement expanded, these guidelines were widely disseminated. A growing number of communities worldwide have implemented local changes with the common vision of making great places to grow older in, thereby helping their older residents enhance daily living experiences and achieve healthy aging (4–6). More Age-Friendly Communities (AFCs) are emerging, which are designed to support healthier living, mobility, independence, safety, and inclusion for older adults at the community level by adapting and changing the urban environment (7). By 2023, the WHO’s Global Network of Age-Friendly Cities and Communities had accredited 1,445 cities and communities across 51 countries, collectively impacting a population exceeding 300 million people globally (8).

Currently, research on the following issues has been increasingly explored in the literature: what constitutes AFCs (e.g., inclusive housing, transportation); why AFCs matter (e.g., they can improve the mental and physical health of older adult and enable rapid response interventions to assist them in addressing significant risks); how to operationalize AFCs (e.g., through favorable policies, conducive programs and measures); how to evaluate AFCs’ achievement (e.g., construct an indicator system to record AFCs’ progress); and what factors facilitate or hinder AFCs (e.g., external resources, local resources, and engaged local participants) (9–13). Nevertheless, due to regional differences, not only do some monitoring and evaluation systems often fail to capture broader community dynamics, but also some age-friendly policy frameworks and effective initiatives lack cross-community scalability (14). Thus, significant gaps remain in knowledge with respect to questions of how to work toward environmental and systemic change at the community level, to better meet people’s needs as they grow older and to allow for flourishing in later life. As stated by Greenfield and Buffel (12), particularly underdeveloped is systematic, contextually grounded research on AFC initiatives in diverse geopolitical, cultural, and economic settings. Fulmer et al. (15) noted that more local knowledge and evidence are needed on how the physical and social environment can be improved in a coherent manner to affect the health and well-being of older adults and other people in the community.

Existing research has documented the progress, challenges and key priorities of enhancing community age-friendliness in some countries (16), including the United States (17), Canada (18), the United Kingdom (19), Australia (20). Unfortunately, a critical gap emerges: the majority of existing studies on AFC initiatives are predominantly based on projects in developed North American and European countries, as these nations with more economic resources were first in embracing the age-friendly agenda in their urban policies and community practices (21, 22). By contrast, studies on AFCs set in Asian contexts remain remarkably limited. This scarcity stems from three key factors. Firstly, the progression of age-friendly initiatives is much slower in these countries, and researchers started relatively late in this field (23). Secondly, the continuous deepening of aging has triggered the rapid emergence and iteration of diverse, confusing concepts related to age and older adults, which have distracted the accumulation of age-friendliness-focused research in the region. Thirdly, while some researchers have begun to explore age-friendly research grounded in Asia, their studies tend to neglect both the local applicability of the WHO-developed age-friendly framework and the practical complexity of advancing age-friendly initiatives within these contexts. These studies primarily adopted quantitative methods and developed assessment tools grounded in the WHO-developed age-friendly framework for cities and communities to evaluate the age-friendliness of specific regions or communities; subsequently, they seek to translate these evaluation findings into concrete recommendations for improvement and actionable projects (24, 25). However, as Jian et al. (26) have demonstrated, the current age-friendly framework is mainly derived from low-density residential contexts in Europe, and thus exhibits considerable limitations when applied to building AFCs in Asian settings characterized by higher residential density. Differences between Asian economies and Western developed countries in terms of housing and care provision for older adults, priorities for advancing AFC development, and established institutional frameworks deserve greater attention (27). Meanwhile, relying solely on quantitative studies of age-friendliness evaluations remains insufficient to capture the sustained efforts and complex dynamics of AFCs in many Asian countries. In fact, given the differences in ideology, developmental stage, societal norms, and priority goals between many Asian nations and Western countries, the age-friendly theoretical framework has inevitably encountered implementation gaps in its localization (28). This has resulted in the development of diverse models for AFC development in practice settings. These subcategories have yet to be adequately identified and elucidated. This gap masks the complexity of AFCs within Asian contexts and hinders the formulation of more effective and context-specific strategies for advancing age-friendliness at the community level.

Consequently, many scholars such as Tan et al. (29) have advocated for extending the discourse on age-friendly practices of Asian counties and cites. Especially, some Asia regions, developing nations, and resource-scarce areas where older adults encounter significant financial, material, social, and other vulnerabilities, have actively engaged with age-friendly initiatives, yet the evidence of AFC development in these settings is either not well established or even absent (30, 31). This insufficiency undermines the Global Network of Age-Friendly Cities and Communities’ capacity to foster inclusive urban aging worldwide. Simultaneously and notably, Asia will become home to the world’s largest aging population, which will cause profound socio-economic changes; most developing countries and resource-constrained areas with high vulnerability for older adults remain ill-prepared to address the systemic transformations necessitated by this demographic shift. This gives these nations and countries a common aspiration to achieve age-friendly goals through low-cost, high-speed and efficient, high-resource-utilization approaches. Against this backdrop, it is vitally necessary to identify evidence-based and context-specific priorities of AFC development for Asian regions, developing countries and resource-scarce areas.

As a key member of the Asian cultural sphere and the developing nation cohort, China confronts an urgent demographic challenge: a rapidly accelerating aging population, which will persist as a structural societal feature for the foreseeable future. By the end of 2023, people aged 60+ constituted 21.1% of the total population (nearly 300 million), with the 65+ cohort accounting for 15.4% (nearly 220 million) (32). Projections from China’s National Health Commission (NHC) indicate that by 2035, people aged 60+ will constitute over 30% of the total population (exceeding 400 million), marking the nation’s entry into a period of hyper-aging. In response to this demographic transformation, China has taken proactive measures. Unique implementation approaches to age-friendly initiatives have been established. A series of policies have been introduced, such as *Guiding Opinions on Promoting Livable Environment for Older Adults*, *Technical Guidelines for Age-Friendly Community Development*, and *National Demonstration Age-Friendly Community Initiative* (33). In China, subnational governments, commercial groups, and social organizations have been activated at the community level to conceive and develop more livable AFCs, just as many areas worked to meet the rapidly increasing needs of older adults during emergencies (34–36).

In contemporary Chinese urban development, a number of communities either self-proclaimed or officially designated as “age-friendly” have emerged, which attempt to become the models of AFC development. These innovative models are broadly categorized into two types based on morphological characteristics and demographic composition: Gated Retirement Communities (GRCs) and Open Multi-Generational Communities (OMGCs). GRCs are physically demarcated by tangible barriers, and implement strict age-segregated strategies (requiring residents to be typically 50+). Their facilities and services are specially designed for the needs of older adults, spanning across the different stages and settings of independent living (IL), assisted living (AL), and nursing care (NL), aiming to create living environments for them (37). In contrast, OMGCs adopt the principles of openness and sharing (both residents and non-residents can use facilities and services), avoiding physical obstacles and access controls. Additionally, these neighborhoods implement age-integrated strategies (encouraging age-diverse residents to live together and providing supporting facilities and services for all age groups), fostering intergenerational interaction. Their facilities and services systems emphasize universal and age-friendly design, intergenerational integration, and harmonious sharing. These two models present distinctive practices of promoting age-friendliness in line with China’s actual situation, contributing to livable and inclusive urban development. However, there are also some challenges that make the realization of “age-friendly” goals more difficult.

This study employed a multi-case study approach to examine the practices of two distinct community types in China and the barriers existing in the realization of “age-friendly” goals. The objectives were to report on the progress of age friendly work in China and propose some insights to bridge the implementation gap of age-friendly initiatives. On the one hand, the evidence provided in this research can help fill the gaps in existing literature and serve as an important window for more regions and organizations to understand the progress of age friendly work in Asian and other developing countries. On the other hand, it can also offer useful experience and practical enlightenment for developing countries, areas with similar aging stages to China and Asian regions with comparable cultural backgrounds in developing AFCs.

## 2 Analytical framework

The WHO age-friendly city framework comprises eight focal points: outdoor spaces and buildings, transportation, housing, social participation, respect and social inclusion, civic participation and employment, communication and information, and community support and health services (38). However, scholars and institutions from diverse cultural and socioeconomic contexts have interpreted AFC frameworks in varying dimensions and focuses (15, 30, 39–41). For instance, reports from British Department for Communities and Local Government concentrated on highlighting social environmental factors such as resident empowerment, healthcare accessibility, lifelong learning opportunities, social cohesion, and adaptive housing (42). Black and Jester (43) prioritized significant health benefits associated with built community features such as housing, transportation, and outdoor spaces and buildings, as older Americans overwhelmingly reported their desire to age in place and in the communities in which they live. Black and Oh (44) contended that AFCs are characterized by catalyzing multiple sectors of society to improve the places and spaces where people reside and interact across the built (e.g., housing), social (e.g., respect and inclusion), and service (e.g., health care) environment, thereby enacting broad-scale social change in geographically-defined municipal settings. Keyes et al. (45) and Warner and Zhang (46) found that AFCs require action among all sectors of society (i.e., public, private, and civil); governments play pivotal roles in, for example, land use planning, provision of supportive services, and enactment of relevant public policies, to facilitate private sector involvement.

A growing body of research from different perspectives has demonstrated the importance of intergenerational interaction opportunities, harmonious communication exchanges, and friendly intergenerational relationships lately (47). Han et al. (48) highlighted the beneficial impacts of intergenerational infrastructures and multi-generational housing models from the perspective of space production, noting that these can ensure spatial equality between generations and enable older residents to maintain the closest social support relationships with their children and younger generation within communities. Fowler Davis et al. (49) investigated the association between social engagement and cognitive frailty among older adults, aurging that communities actively encouraging intergenerational interaction tend to reduce isolation. Ermer et al. (50) and Kwong and Yan (51) analyzed how intergenerational programs involving collaboration, shared learning experiences, and interactive understanding alleviate ageist attitudes among young people, build empathetic and supportive relationships with older adults, and bridge divides across generations. Tohit and Haque (52) proposed the establishment of a cohesive culture that values contributions from all age groups, with the perspective that younger generation can play a role in addressing the challenges of an aging society. The inclusive approach can support younger and older generations to thrive together, enhancing social cohesion and collective well-being. This is shifting AFCs’ focus away from older adults to one where social and physical facilities mutually beneficial to all persons, regardless of age (30).

Furthermore, sustainability is integral to the development and implementation of age-friendly initiative, serving a distinct purpose in the successful continuation of AFC programs (53). In the absence of sustainability, these age-friendly projects and plans risk stagnation and failure, not only breaching commitments to the target population but also squandering public resources (54). More pressingly, with many countries experiencing economic austerity and numerous priorities competing limited resources, the implementation of AFCs is intertwined with multiple pressures, facing acute challenges in vulnerability and sustainability (16). Policymakers, researchers, and community practitioners have raised important concerns about uncertainties around AFC sustainability. However, research on the problem of implementing sustainable, long-term age-friendly initiatives remains limited (55). Thus, there is an urgent need to explore factors that facilitate or hinder AFC sustainability. While existing literature has defined sustainability and categorized it into economic, environmental, and social dimensions (56), some scholars have further developed this framework by building on the concepts of sustainability and integrating the characteristics of AFCs. Specifically, in AFC research, sustainability is generally defined as the duration of program lasting, the capacity to maintain community viability, service delivery, and plan implementation, and the degree to which initiatives become permanent and institutionalized beyond initial development (36).

To sum up, as reflected in empirical studies and theoretical frameworks, scholarly understandings on AFCs differ. However, by reviewing gerontology, urban planning, and social gerontology literature (15, 39, 40, 57–60), it can be well understood that policies, facilities, services, intergenerational relationships, and sustainability are included as the key elements to enhance age-friendliness of community. Base on this, this study has established an analytical framework incorporating the above elements. Meanwhile, contextualizing these dimensions within the socioeconomic and cultural milieus of Asian societies has further validated the framework’s applicability. First, populations in Asian countries are aging at a faster pace compared to those in European and American countries. Thus, there is an urgent need for policies, facilities and services in age-friendly environments to systematically adapt to emerging demographic and social transformations. Second, the culture of filial duty (respecting and caring for parents and older adults) constitutes a core value in Asian societies (61). With the passage of time and society changes, the expressions of respect for older adults are changing, yet the core ethical principles centered on intergenerational reciprocity and emotional bonds remain stable (62). This cultural gene still profoundly influences societal development in these countries (63). Third, sustainable development is a global priority, particularly critical for Asian nations and those in the extensive phase of socioeconomic development to balance present needs with the well-being of future generations (64). This also equally applies to the development of AFCs in these countries.

According to the above, an analytical framework embedded within the Asian context has been developed to systematically document and identify the age-friendly efforts at the community level in China as well as some other Asian countries (see Table 1).

**Table 1:** An analytical framework of community age-friendliness.

Elements	Descriptions
Policies	Get a series of policies and public funds from the government, to provide necessary resources for AFCs and attract multi-stakeholder to participate.
Facilities	Build a physical spatial environment and service infrastructure to meet the needs of older adults and continuously support age-related changes (e.g., accessible housing, age-adaptive outdoor environments, healthcare facilities, hospitals, supermarkets, community centers, parks).
Services	Cover diversified services for older adults (e.g., housing maintenance and renovation, basic medical services, family-based and community-based health care, leisure and entertainment), as well as various services for other age groups.
Intergenerational relationships	Investigate intergenerational interaction among older adults, youth, children, and other generations and establish non-discriminatory relations in the community.
Sustainability	Possess the capability to deliver services, sustain, survive, and thrive in order to maintain age-friendly commitments and prevent plan from reduction or interruption.

## 3 Method

### 3.1 Background

This study employed a multi-case study method, which allowed us to intensively examine the progress of AFCs in China in a rich, detailed, complete, and rigorous manner, and to identify existing systemic gaps and effective implementation measure of achieving “age-friendly” goals (36, 65). This method offers obvious advantages over single-case designs. It allows researchers to deeply explore and explain the “how,” “what” and “why” of complex problems in real life from various dimensions (16, 66). By supporting researchers in collecting data from multiple cases and conducting horizontal comparison and vertical analysis, this method brings new possibilities for acquiring new knowledge and improving research quality. It has shown to be particularly instrumental in comparing the implementation and the development of age-friendly programs across different communities (67, 68). Meanwhile, it is worth noting that this study adapted a non-interventional methodology. Its objective is to conduct research through observing and recording naturally occurring data and phenomena, without active intervention in any behaviors, states, or environments, thereby enabling researchers to more objectively and comprehensively capture the actual dynamics of facility and service utilization, as well as relationship-building, within GRCs and OMGCs.

Guided by this methodological framework, three Gated Retirement Communities (GRCs) and three Open Multi-Generational Communities (OMGCs) were selected in Jiangsu province, China for case analysis. This section elucidates the reasons for selecting Jiangsu as the case setting. In terms of geographical location, Jiangsu is positioned in the central region of China’s eastern coast, at the lower reaches of the Yangtze and Huaihe Rivers, playing a pivotal role in the Yangtze River Delta megaregion. In terms of economic growth, Jiangsu has always had outstanding performance. In 2024, its regional GDP reached CNY 13.7 trillion, ranking second among all provinces in China; its economic growth rate marked a 5.8% year-on-year increase, ranking first among all provinces in China. It indicates that Jiangsu has active social capital which provides more favorable development conditions for AFCs. In terms of demographic development, Jiangsu is both a populous and rapidly aging province. By the end of 2024, it had a permanent population of 85.26 million; the population aged 65+ in Jiangsu had reached 15.94 million (18.7% of its total), which was 3.1 percentage points higher than the national level (15.6%). Significantly, Jiangsu is the first province in China to enter the aging society. In this context, Jiangsu has attached significant importance to fostering supportive physical, service and social environments for older adults, and implemented proactive policies and measures to address the surging silver wave. The work primarily includes: (1) Introducing policies to support in terms of planning, land use, funding, and utility provision (water and electricity), to encourage and guide diverse social stakeholders to collaboratively develop age-friendly physical environments and service facilities; (2) Investing in community infrastructure renovations (e.g., road upgrades and power supply optimization), constructing barrier-free facilities (e.g., ramps, handrails, public restrooms, elevators), and adding/retrofitting community service amenities (e.g., supermarkets, medical clinics) to enhance living quality and accessibility for older adults; (3) Constructing age-adaptive residential communities with comprehensive infrastructure and service systems, centered on home-based and community-based supports, to offer healthy, comfortable living environments and practical services; (4) Improving in-home living environments embracing “toilet-bath safety, indoor mobility convenience, living environment enhancement, intelligent monitoring, and assistive device adaptation,” providing age-adaptive renovations for families with urgent needs. Consequently, Jiangsu has remained at the forefront of AFCs, which makes Jiangsu an exemplary location for studying AFCs in the Chinese context.

### 3.2 Sampling

**Figure 1:**
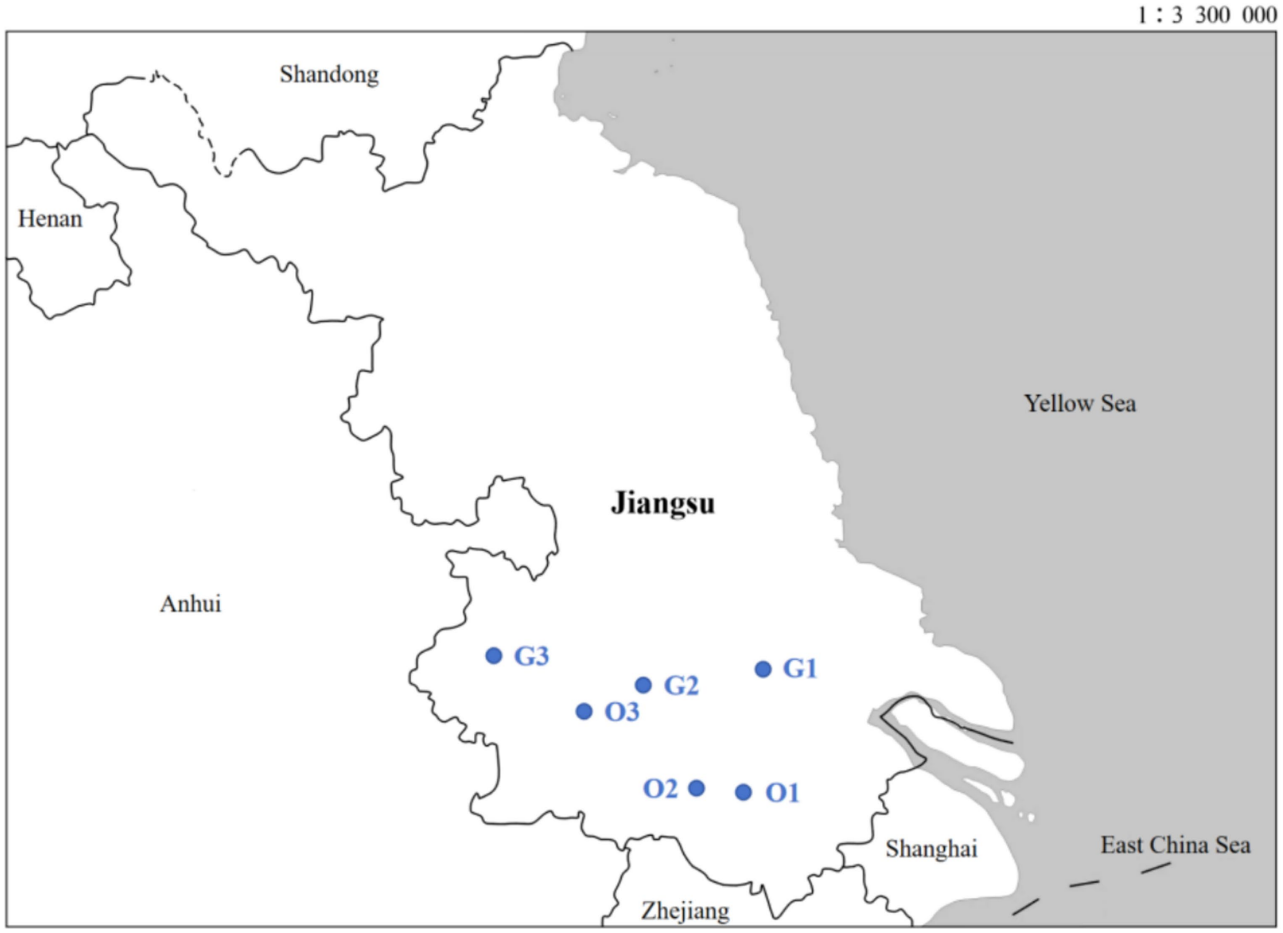
Map of selected AFCs.

To secure representative samples, this study used a purposeful sampling strategy to identify research cases. This process was structured into three distinct phases, each underpinned by a set of selection and classification criteria. In the initial phase, the selection criteria for cases were: (1) These communities had recognition of age-friendly attributes through self-certification or official accreditation; (2) They were operational for a period of time, to ensure abundant data to track the progression of age-friendliness; (3) They implemented age-friendly initiatives (containing but not limited to age-friendly environment construction and supportive service provision). This stage aimed to establish the sample pool of AFCs in Jiangsu. The second phase involved a hierarchical classification of the identified AFCs. (1) According to the category, the neighborhoods in the sample pool were bifurcated into Gated Retirement Communities and Multi-Generational Communities. (2) Within each subgroup, samples were further stratified based on spatial scale, thereby preventing the neglect of small-scale AFCs and ensuring representation across categories. In the final sampling phase, the selection criteria emphasized: (1) Data accessibility and availability were the primary factors to be considered; (2) Inclusion of both newly constructed and retrofitted AFCs to capture more information of China’s AFC development; (3) Inclusion of geographic diversity, covering both urban cores and outskirts, to mitigate sampling bias. Ultimately, six eligible cases, as depicted in Figure 1, were selected. To safeguard objectivity and mitigate potential conflicts of interest arising from commercial affiliations, sample identifiers were anonymized, ensuring strict confidentiality throughout the study.

G1, commissioned in 2008, spans 72,666 m^2^. It is a purpose-built community for older adults initiated by municipal authorities with foreign investment. Comprising three high-rise apartments, 10 luxury villas, and 14 mid-rise buildings (500 residential units), it integrates nursing facilities, cultural centers, rehabilitation zones, lifestyle service departments, and commercial precincts, offering a comprehensive hub for living, recuperation, rehabilitation, healthcare, education, recreation, and commerce. Targeting active, semi-disabled, and disabled older adults, it delivers holistic support from daily care and leisure to medical nursing and long-term care, with a capacity of 1,000 residents.

G2, founded in 2011 and operational since 2014, is a 177,000 m^2^ integrated living community endorsed by the civil affairs bureau and developed by commercial entities. Guided by the principles of “dignity, modernity, style, and ecology,” its mission is to “honor filial piety, enable older adults well-being, and alleviate government burden.” With 1,789 residential units and 90,000 + m^2^ (building area) of functional spaces (medical, cultural, commercial, and recreational facilities), it accommodates 4,000 planned residents (3,000 currently enrolled, mean age 77), supported by 350 staff. This neighborhood has received official accreditation as a “Model Age-Friendly Community.”

G3, commissioned in 2019, is an urban community spanning 120,200 m^2^ (180,000 m^2^ built area), featuring 125,000 m^2^ age-adaptive housing, 11,000 m^2^ club facilities, 14,000 m^2^ nursing homes, and 30,000 m^2^ rehabilitation hospitals (5,000-resident capacity). Blending Chinese “home” and “courtyard” cultures with Suzhou garden aesthetics and deinstitutionalized living, it creates diverse spatial experiences. Upholding age-friendliness and active aging, its six service domains----daily care, health management, medical nursing, nutrition, rehabilitation, and cultural activities----cater to comprehensive needs of older adults.

O1, commissioned in 2017, spans 54,000 m^2^ and comprises over 30 residential buildings, of which only 6 are purpose-built age-adaptive homes. Incorporating 188 barrier-free and gerontechnology designs, the community enables older adults at varying health and self-care stages to maintain independence in a safe, accessible environment, thereby enhancing late-life quality. Embracing intergenerational living, it features shared activity hubs, affordable canteens, bakeries, supermarkets, laundries, a swimming pool, rooftop farm, and rehabilitation hospital, catering to daily needs and professional care for all age groups.

O2, located in the central business district, is a 30-story vertical complex covering 16,540 m^2^ (with a built area of 60,000 m^2^), including 18,000 m^2^ of amenity-rich spaces. Facilities include a health care center, dental clinic, coffee shops, affordable/eatery restaurants, specialty dining, banquet halls, a flagship bookstore, Su-style art gallery, photography club, print/baking/culinary workshops, whisky-wine lounge, children’s art academy, art boutiques, fashion conveniences, high-end tailoring studios, medical aesthetics clinics, hair-SPA wellness centers, Putiyan Fitness Club, heated pool, and multi-court sports hall. The neighborhood offers facilities and service accessible to all age.

O3, originally constructed in 2000, became one of the urban renewal projects funded by the municipal government’s livelihood improvement initiative in 2022 and completed renovation in 2023, addressing aging demographics and infrastructure decay. The renovation included accessible upgrades, infrastructure improvements, the addition of care amenities and intergenerational spaces, facade/road revitalization, lighting-greenery enhancements, and recruitment of professional service providers. Now, it hosts a home-based care center, a 365-grid service station, neighborhood hub, library, pocket parks, and childcare center—evolving into an inclusive AFC that fosters support, participation, and development for all ages (Table 2).

**Table 2:** A concise overview of each case.

Type	Gated retirement communities (GRCs)			Multi-generational communities (OMGCs)		
Case	G1	G2	G3	O1	O2	O3
Construction mode	Newly constructed	Newly constructed	Newly constructed	Newly constructed	Newly constructed	Renovated and upgraded
Year of completion	2007	2014	2019	2017	2020	2023
Floor space (m^2^)	72,666	177,000	120,200	54,000	16,540	50,000
Planned capacity	1,000	4,000	5,000	2,200	800	1780
Location	Urban core	Outskirts	Outskirts	Outskirts	Urban core	Urban core
Land use nature	Medical and care attributes	Medical and care attributes	Medical and care attributes	Residential attributes	Commercial attributes	Residential attributes

### 3.3 Data collection

The formal research was conducted from March 2024 to November 2024, preceded by a multi-year pre-survey. The pre-survey, which commenced in 2015, was centered in Jiangsu Province with a focus on age-friendly practices, led by the second author—a scholar of public policy analysis and gerontology. Through years of field observations, the second author amassed extensive first-hand and second-hand data, building friendly relationships with operators of AFCs. These communities provided the studies with the necessary information, which established a robust foundation for sample selection and data collection. Previous studies have shown that data for the case study were collected from multiple sources, including textual evidence, site observations, which are available to provide a clear account of the case in question (69, 70). To develop an in-depth understanding of age-friendly measures in GRCs and OMGCs in China, this study employed various data collection methods.

Firstly, this study established a robust foundation for defining the analytical framework and bridging it with practical applications by systematically searching academic literature databases, attending disciplinary conferences, and reviewing relevant research reports, papers, commentaries, and publications. The pre-established systematic analytical framework directly guided the data collection process, enhancing the targetness and efficiency of information gathering. Secondly, government portals were scoured to procure regional socioeconomic data, government work reports, age-friendly policy documents, public-private partnership agreements, and commendation announcements, aimed at tracking evolutions in policy implementation and municipal resource allocation trends for age-friendly initiatives at the community level. Thirdly, community official websites and work briefings were leveraged to obtain detailed profiles (including geographic location, spatial dimensions, facility inventories, service portfolios, land use typologies), progress updates, promotional materials, notices, complaints, and conflict mediation records, enabling researchers to understand community development trajectories, service priorities, facility utilization patterns, and existing contradictions. Fourthly, to mitigate information bias from official channels, news media reports (particularly critical analyses) were collected to uncover key events, illuminating vulnerabilities, unfulfilled commitments, and sustainability risks in community development. Finally, on-site inspections combined with observations, notetaking, and photography were conducted to collect case study data. Researchers posited that firsthand fieldwork was indispensable for penetrating information barriers, identifying performative elements in textual materials, and mapping discrepancies between policy discourse and on-the-ground practices. However, to reduce information bias introduced by the intervention, researchers neither engaged with residents nor accessed personal privacy information or specific behavioral details of individual residents. Instead, the study prioritized observing “group behavioral patterns” (e.g., the frequency of public facility utilization) to ensure the acquisition of first-hand data in order to achieve research objectives. Data collection ceased when thematic saturation was achieved, i.e., no new concepts, perspectives, or events emerged.

### 3.4 Data analysis

To analyze research questions within the established analytical framework, this research adopted a directed content analysis method. The method is particularly suitable for contexts with predefined analytical directions and categorical systems, enabling systematic data analysis while avoiding the blindness in the analysis process (71). Therefore, upon acquiring textual and visual data, each researcher documented preliminary observations and assigned thematic categorizations, engaging in regular debriefing sessions with co-researchers. Leveraging these initial insights, both researchers independently developed preliminary coding schemes after data collection. Prior to formal analysis, a cross-reading exercise of these schemes was conducted, systematically documenting areas for refinement. The formal data analysis adhered to a four-phase. First, the research scope was reaffirmed by examining the advancement and bottlenecks of China’s two AFC models, aiming to distill effective implementation strategies. Second, a process of close reading, comparative analysis, and careful examination was applied to all collected materials and field notes, enhancing data clarity, identifying programmatic nuances, and deepening case comprehension. Third, data were taxonomically organized according to the established analytical framework. The researchers commenced by jointly reviewing the preliminary coding schemes and identifying gaps, followed by independent text coding. Through iterative cycles of collaborative reflection, a finalized coding manual was developed, with convergent codes aggregated into sub-themes and overarching themes (Table 3). Fourth, interpretive and thematic analyses were employed, involving joint conceptualization and analytical discussions. Regular symposiums were convened to resolve disagreements. After a several-months-long iterative and inclusive process, disagreements were eliminated, mutual agreement was reached, and findings and conclusions were confirmed.

**Table 3:** Themes and subthemes.

Themes	Subthemes
Policies	Direction of public resource allocation (support for planning, land use, water consumption, electricity use, etc.; subsidies for project construction and operation; direct investment of public funds)
	The emergence of inequity
Facilities	Resource portfolio and homogeneity
	Current focus of the community
	Facility and resource utilization efficiency
Services	Service contents and service recipients
	Hidden service contradictions and conflicts (e.g., increasing service fees vs. rising service costs, impairment of older adults’ rights and interests, etc.)
Intergenerational relationships	Measures for age exclusion and age integration
	Intergenerational communication and interaction patterns
Sustainability	Long-term operational status of the community (overall operation, and the operation of each component such as hospitals, nursing homes, recreational facilities, etc.)
	Challenges of vulnerability and uncertainty
	Integration into urban development

## 4 Findings

This study examined how these two types of Chinese communities promote age-friendliness in terms of policies, facilities, services, intergenerational relationships and sustainability, while also analyzing persisting challenges.

### 4.1 Policies

In the early 21st century, as the Chinese government initiated the socialization of care services, GRCs effectively utilized its own capacity to meet the specific needs of old adults, aligning with the national strategy of “attracting social forces in the construction of the care service system.” These characteristics enabled them to secure preferential policies in areas such as land application and financial subsidies. For instance, G1 obtained the right to use approximately 70,000 m^2^ of land at significantly discounted prices below the market average. Meanwhile, it received public funding supports during the construction phase and expedited approvals for qualification acquisition in development and sales. Additionally, it enjoyed exemptions on municipal comprehensive fees and other related expenses.

However, such government investments in GRCs have inadvertently exacerbated spatial injustice and social inequality. Many low-income older adults who genuinely needed government assistance were unable to access the support provided by these facilities and services in GRCs. Specifically, they faced dual exclusion: their economic capacity did not meet the community entry thresholds, and these communities employed effective physical exclusion mechanisms to restrict their access. The arrangement violated the justice principle of “prioritizing the most disadvantaged groups” in public resources allocation.

As awareness of responsibilities and boundaries grew, the Chinese government recognized this reality. Government investments in “segregated urban spaces” such as GRCs, which offered elegant environments, comprehensive facilities, and high-quality services for older adults, risked becoming selective welfare for upper-middle-class older groups, thereby intensifying imbalances and inequities. In response, the government reduced policy supports for this type of communities. Consequently, the preferential policies received by GRCs were decreased, and so was OMGCs. For example, O2, established in 2020, not only adopted commercial land use but also implemented commercial pricing for water and electricity consumption.

Since 2016, with the housing market structure evolving into an existing stock-dominant stage (72, 73), age-friendly initiatives in Chinese communities have entered a new phase. The government has placed significant emphasis on renovating and upgrading existing mixed-age neighborhoods characterized by dilapidated facilities, inadequate age-friendly and universal design, safety oversights, and service deficits. To address these gaps, renovation programs started in some communities, like O3. It renovated roads, walls, greenery, streetlights, etc., and added facilities such as rehabilitation and nursing centers, comprehensive housekeeping service centers, children’s playgrounds, intergenerational reading rooms, and all-age learning spaces. These improvements aimed to provide convenience and supports for residents of all age groups within and around the community. The government provided corresponding policy supports, including direct investment in construction funds, operational subsidies for facilities, and targeted procurement of public services in this process.

This age-friendly approach, designed to benefit all ordinary older groups and all-age groups rather than just upper-middle-class older groups, is currently being widely adopted across Chinese communities. Supported by government resources, it can more effectively enhance public fund utilization efficiency while advancing social equity.

Overall, in contrast to investing in newly constructed GRCs, providing policy frameworks and financial incentives to support OMGCs, especially for the renewal and addition of age-friendly content in existing mixed-age neighborhoods, represents a more effective, efficient, and equitable approach to advancing age-friendliness at the community level.

### 4.2 Facilities

Residents experience greater perceived supports and facilities achieve greater utilization efficiency in communities with enhanced age-friendliness (74).

The GRCs aimed to create living environments enabling older adults to fulfill their aspirations for quality life. Their facilities were extremely rich, but showed a high degree of similarity. For instance, both G3 and G2 featured over 30 functional spaces for older adults to exercise, play, communicate, study, pray, relax, eat and recuperate, in addition to age-adaptive housing. However, these facilities exhibited low utilization efficiency and limited supportive capacity. A survey conducted in G2 revealed that only 10% of residents regularly used functional amenities, primarily attributed to three factors: (1) although most residents were in independent living stages, their average age exceeding 70 years led to preferences for quiet lifestyles centered on home-based activities like chatting and watching television; (2) facilities such as gateball courts mismatched generational hobbies and habits formed during their youth and adulthood; (3) this gated model restricted access for external users, resulting in residents in the community not utilizing the resources, and consequently, no other individuals made use of them either. There were inevitably significant wastes of resources, which violated the principle of “intensive utilization of urban resources.”

In contrast, OMGCs adopted facility configurations based on market research and regional needs, while maintaining open access beyond internal residents. This approach fostered diversified facility portfolios and optimized usage rates.

Consider O2, which integrated facilities for all age, including children’s playgrounds, beauty salons, postpartum care centers, sports fields, age-adaptive houses, nostalgic museums, canteens, and supermarkets. They emphasized that age-friendliness required not just specific provisions for older adults but also inclusive facilities capable of enhancing interactions in family. For example, female older adults can go to the beauty salon with younger female family members, while grandparents accompanied grandchildren to play in children’s playgrounds. Meanwhile, through rigorous needs assessments in this district, J ensured that the facilities were appealing to surrounding residents and served as preferred local resources when needed. Therefore, facilities within OMGCs expanded their supportive capacity and enhanced their utilization rates.

This paper highlights a critical yet often overlooked principle in AFC design: age-friendliness and facility supportiveness do not hinge on an excessive amenities, but on the foundation of understanding residents and service users, identifying their urgent needs and delivering tailored provisions. The mindless replication of extensive facilities fails to advance age-friendliness.

### 4.3 Services

The GRCs and OMGCs have both relied on diversified facilities and professional service teams to enhance age-friendliness by providing comprehensive and caring services for residents. For instance, G2 assigned dedicated service personnel to provide butler services for dozens of households in each building. O2 offered a comprehensive range of services, including nutritious meals, cultural and recreational activities, health management, sports rehabilitation, and professional nursing. Residents can enjoyed these services without leaving the community.

However, the capabilities of the two types of community services exhibited substantial differences. Represented by O2 and O1, OMGCs were anticipated to generate stable revenue from service operations from the outset. Therefore, they adopted a sustainable revenue strategy by implementing a user-pay model for most services through revenue generation from both internal residents and external users, while maintaining free access of some common areas. This approach ensured financial stability and service continuity.

In contrast, GRCs encountered challenges in service provision, particularly an increased likelihood of service reduction or the imposition of additional service fees. Due to the majority of their facilities and services being provided free of charge to residents, combined with physical barriers and security protocols excluding non-residents, GRCs struggled to generate stable operational income. This revenue gap often led to the destruction of original service commitments, when operational costs exceeded limited revenues.

The G1 case illustrated this vulnerability. The commercial group refused to reinvest the profits they had already obtained in hand into the later operation due to the huge financial burden of maintenance costs and service expenses. Specifically, the service agreement signed with the residents explicitly stated that residents did not need to pay for maintenance costs and service expenses at that time. The annual maintenance costs of several hundred thousand (CNY) for the residential buildings were to be covered by the commercial group, which had committed to providing this service. Furthermore, the commercial group also pledged to deliver long-term professional services. However, this investment proved to be akin to a “bottomless pit” with little profits. As time progressed, both the cost of maintenance and the cost of service provision continued to increase exponentially. Consequently, the commercial group choosed to withdrew from the community, breaching their commitments for the government, society, and older adults and triggering a series of contradictions and conflicts.

Similarly, yet distinctly, G3 resorted to imposing monthly service fees (ranging from CNY 2,500 to 9,500) on top of the CNY 1.3 million entrance deposit to ensure service provisions. This approach contradicted the commitments previously made to residents. As a result, many residents have voiced significant dissatisfaction and some have even opted to leave the community.

In China, the implementation of age-friendly initiatives has existed for a period of time. Initially, vigorous infrastructure expansion was prioritized as the cardinal task. Consequently, the provision of supportive facilities for older adults experienced a substantial surge within a short timeframe. Nevertheless, persistent low facility utilization has increasingly emerged as a critical issue. To this day, the overemphasis on infrastructure rather than services remains a pronounced challenge in China’s age-friendly practices. Many AFCs, such as the above-mentioned G1 and G3, attracted older adults with rhetorically appealing service commitments. In reality, however, their focus remains myopic, as services have never been elevated to a position of strategic importance. At the community level, implementing age-friendliness necessitates placing services at the vanguard of initiatives.

### 4.4 Intergenerational relationships

Age-Friendly Guidelines note that robust social networks and intergenerational interaction are fundamental to the well-being of older adults. GRCs and OMGCs presented distinct characteristics in intergenerational interaction patterns due to divergent conceptual frameworks and operational strategies.

The GRCs prioritized creating segregated, secure living environments for older adults through systematic age-restriction mechanisms. For instance, G1 conducted annual resident screenings to maintain a minimum age threshold of 60 before commercial groups withdrew. Individuals below this threshold were gradually cleared out of the community. These neighborhoods focused primarily on intra-generational socialization with limited intergenerational interaction, despite family visitations where family members accompanied older adults to live, stay overnight and eat being encouraged. This was because, within these communities, young family members faced restrictions on their participation in activities and use of facilities. For example, in G2, middle-aged and young people as well as children were prohibited from joining more than 40 kinds of interest clubs; higher fees were imposed on younger individuals for using community facilities to effectively discourage their usage.

Spatial and temporal dynamics in these settings often produced age-based social exclusion. Residents of G3, for instance, expressed antipathy toward external children and youth, citing safety concerns and resource competition. Children were loud and liked running and jumping, which posed safety risks; Young people’s facility usage increased waiting times. Residents argued that since these were homes for older adults, young people should leave. Physical and cultural boundaries reinforced the narrative of “older-only” spaces, institutionalizing intergenerational segregation.

Conversely, OMGCs adopted inclusive models to expand older adults’ social connectivity, enrich their social experience, and enhancing intergenerational exchange. O1 typified this model by maintaining permeable boundaries and shared common areas for residents and non-residents, supporting diverse age-inclusive activities such as dining, cultural events, and recreational programs. These initiatives increased spontaneous interactions among internal residents, external users, and different age groups. H believed that inclusive environments and continuous engagement with varied age cohorts were beneficial for enhancing the psychological health and well-being of older adults, embodying the true essence of age-friendliness.

The above case demonstrated that over-prioritizing a specific age group is likely to induce age-based social exclusion, thereby exacerbating intergenerational tension and conflicts. This will run counter to the core tenet of fostering non-discriminatory intergenerational dynamics within age-friendly initiatives.

### 4.5 Sustainability

Sustainability is an integral component of age-friendly frameworks. In this study, it was concretized as the capacity for resilience and continuity, maintaining commitments and ensuring age-friendly initiatives remain unimpaired by resource reductions or operational disruptions.

The GRCs attempted to establish self-circulating internal ecosystems, yet empirical evidence revealed their unsustainability. The reason was that infrastructure such as hospitals and nursing homes, which required substantial investment, cannot depend solely on intra-community demands for survival. Take G2 as an example. When it was established in 2015, the average age of residents was 67. Now, the average age has increased to 77, yet many residents still did not need hospitalization or nursing home care. To address this, it adopted an “external-first” operational strategy, which prioritized public accessibility before serving internal residents. Without this approach, their financial sustainability would be at risk, ultimately weakening the support capability for internal residents in the community.

When CRCs were compelled to reintroduce underutilized facilities into competitive markets in order to maintain development, they faced enormous crisis. For instance, the rehabilitation hospital in G2 was the largest private healthcare provider in the district, but it lacked a clear competitive edge compared to public institutions. Hence, it continued to operate at a loss annually. Similarly, G3 attempted to repurpose underused recreational facilities in recent years due to development crisis, which have proven arduous and torturous. First, geographically disadvantages of its facilities located in the community center rather than near main thoroughfares struggled to attract external patrons. Second, functional reconfiguration further presented dilemmas: determining what kinds of functions need transformation and provision to meet the needs of internal and external residents for achieving stable development, and how services can be designed to effectively satisfy these needs? These questions will need substantial investment and iterative experimentation to resolve.

In contrast, OMGCs adopted market-oriented approaches from the outset to ensure long-term sustainability. O2 exemplified this strategy through three key pillars: (1) urban core positioning with proximity to dense residential clusters and high pedestrian flow; (2) tailored facility portfolios based on micro-market analysis, prioritizing internal residents while meeting underserved local needs; (3) dynamic service adjustments validated through continuous market feedback. They argued that their ability to provide continuous services for residents hinged on their survival in the market. The adaptive sustainability of OMGCs far surpassed the rigid models of CRCs.

Operating AFCs in a closed manner represents a critical flaw, as such an approach fails to promote sustainable development. Instead, openness and large-scale integration into the urban fabric constitute the key to fostering AFC development.

## 5 Discussion

A variety of effective and efficient age-friendly policy frameworks, action guidelines, and evaluation tools have been developed to achieve “age-friendly” goals more comprehensively, record and compare the progress of age-friendly initiatives across different regions (44, 48). However, some have criticized these frameworks and guidelines for being too Western-oriented, arguing that they might not align well with social foundations and value ethics in Asia (75). To address this gap, this paper has developed an analytical framework of community age-friendliness within the Asian context, which encompassed multiple dimensions including policies, facilities, services, intergenerational relationships, and sustainability. This framework can accurately document the progress of age-friendly initiatives across more Asian countries and developing nations. To further validate the applicability of this framework and identify effective strategies to enhance age-friendliness at the community level, this paper employed the framework to investigate how Gated Retirement Communities (GRCs) and Open Multi-Generational Communities (OMGCs) in China foster age-friendliness. Among them, some key priorities and the remaining challenges were highlighted. Table 4 summarized the progress of age-friendly works in the two types of communities in China as reported in the study.

**Table 4:** The progress age-friendly works in Chinese two types of communities.

Elements	Gated retirement community	Open multi-generational community
Policies	•Benefit from policy supports in the early stage of development including land supply, financial subsidies •Intensify spatial injustice and social inequality and obtain policy support diminished in the later stages	•Less access to policy support in land and hydropower •Obtain policy support of renovation and upgrading in existing mixed-age communities
Facilities	•Specially designed for older adults •Be gated and restrict the entry of outsiders •Equip supporting facilities spanning across the different stages of independent living, assisted living, and nursing care •Rich facilities, high similarity, weak sense of support and low utilization efficiency	•Decentralization and de-specialization •Open, shared and inclusive •Integrated facilities for all age including children’s playgrounds, beauty salons, care centers, age-adaptive houses, ect. •Different facility configurations and higher utilization rates
Services	•Provide comprehensive services for older adults including daily living assistance, basic healthcare, and long-term care •Risks on reduced services or added service fees	•Provide diversified services for all age •Have financial stability and service continuity
Intergenerational relationships	•Systematic age-restriction mechanisms •Focus primarily on intra-generational socialization •Prone to intergenerational exclusion.	•Enhance social connectivity and intergenerational exchange •Beneficial for intergenerational solidarity and integration
Sustainability	•Risks on unsustainability •Huge cost of transformation	•Adopt market-oriented approaches •Have long-term sustainability

Effective and efficient policies are essential to realize communities’ age-friendliness, which can overcome obstacles to the development of AFCs (48). However, the development of AFC policies often encounters various problems (76, 77). Among these challenges, the policy tended to favor wealthy groups who contributed to capital accumulation, while paying limited attention to marginalized groups. This remained a significant obstacle hindering the development of AFC policies (48, 78, 79). As reported in this study, the government misallocated public funds, preferential policies, and other resources to GRCs characterized by dual exclusion mechanisms of spatial segregation and economic filtering, abundant facilities, and high-end quality. This allocation led to the concentration of policy resources among the high-income older adult group, thereby exacerbating social inequality. This has sparked critical inquiries into age-friendly initiatives and associated activities (16), as the flawed orientation of age-friendly policies has failed to substantially address the persistent challenge of many older adults residing in highly vulnerable environments (80). This not only undermines the demonstration of age-friendly benefits but also hinders the scalability of such initiatives across broader community contexts. Against this background, the possibility of AFC policies needs to be innovatively explored. After years of development, age-friendly works in China have undergone a significant shift. The government is now focusing more on the age-friendly development of ordinary, established mixed-age communities, offering financial funds and policy supports to their facilitate their upgrades, renovations, and the addition of age-friendly facilities and services. For instance, the transformative renovations implemented by the O3 within OMGCs. Fundamentally, this approach re-centers fairness and justice as the core principles of AFC policies. Enabling policies to benefit a broader range of older adults and allowing them across various income levels and capabilities to find their appropriate roles and receive supports within the community, aligns more closely with the essential meaning of “age-friendly” (81, 82). These efforts offer valuable insights for implementing age-friendly policy frameworks at the community level in some regions. It is crucial to acknowledge that biases are inherent in public policies, a systemic issue embedded in the policy lifecycle. To unleash the full value of age-friendly initiatives and extend benefits to a broader older adults, public policies must prioritize addressing inequalities in access to services and community resources, particularly for the most vulnerable older adults (31). Implementing urban renewal strategies for ordinary existing established mixed-age communities to achieve broader promotion of age-friendliness is the development direction of age-friendly work.

The AFCs focus on modifying the broader physical environments and service environments of older adults to enhance their capacity to function optimally in their own homes and communities (2, 6). However, this did not mean that all communities have uniformly needed the same facilities and services. This study revealed that despite the extensive range of facilities and services available in GRCs, older adults living in these communities still experienced a low sense of support; while OMGCs offered more robust support to residents of diverse age groups within the community and surrounding populations through differentiated facility configurations and service provisions. The underlying reason was that while numerous facilities and services appeared to be provided, they failed to genuinely address the specific needs of residents. Previous studies have demonstrated that implementing age-friendly initiatives necessitates responsiveness to the heterogeneous needs of diverse older adult subgroups (16). Nevertheless, these studies remained silent on the methodologies employed to identify older adults’ needs. In some communities, needs identification was rooted in theoretical constructs of aging populations, such as GRCs, whereas others, like OMGCs, enhanced needs awareness through rigorous demand-assessment research. These divergent approaches to needs identification are likely to shape distinct trajectories for age-friendly community development. It was crucial to recognize that fostering age-friendly environments hinged on creating spaces that ensured old adults received adequate support. Therefore, the development of AFCs needs to take into account the heterogeneous characteristics of different communities and the diverse needs of their residents. Similar to OMGCs, thorough market research should be conducted, and facility and service plans should be tailored to local conditions and regional demands, thereby strengthening the perceived supportive environment of facilities and services for community residents. Otherwise, it may further exacerbate the sense of deprivation experienced by vulnerable residents in certain communities (83). Meanwhile, this study revealed significant disparities in service capabilities between GRCs and OMGCs, rooted in their divergent approaches to service provision and delivery. AFCs that prioritize service delivery and honor service commitments demonstrate greater operational capability, whereas failure to do so compromises residents’ rights and erodes public trust in AFC initiatives. In conclusion, tailoring facility configurations and service offerings to local needs while emphasizing the fulfillment of service commitments to end-users represents a people-centered and reality-based approach to advancing age-friendly practices at the community level (84).

Whilst some studies have advocated and noted that no design will suit everyone perfectly (85), it was not advisable to focus solely on one group due to the absence of a perfect design. Fang et al. (86) has figured that spaces and places designed to cater for one population group at the expense of another can create schisms across generations. This study has also uncovered intergenerational tensions within GRCs specifically designed for older adults. In these communities, children and young adults were frequently regarded as unwelcome visitors. Children who entered unexpectedly were viewed as potential “sources of danger,” while young adults utilizing community facilities were labeled as “resource appropriators.” Intergenerational exclusion and social isolation driven by age-based discrimination have emerged, which not only undermined the physical and mental well-being of older adults but also contradicts the core principles of AFCs (87, 88). As posited by Rémillard-Boilard et al. (16), tackling ageism and intergenerational exclusion was not only important in improving the quality of life of older people, but also in enabling the delivery of age-friendly programs themselves. When younger and older generations engaged in positive interaction, mutual respect and understanding are fostered, and enhanced intergenerational harmony thereby benefited society in multiple long-term ways (52). Puhakka et al. (89) clarified that closely aligned with the concept of age friendliness is age integration, from which perspective age-friendly living environments can be understood as those in which people of all ages can experience a sense of belonging and supporting. Yin et al et al. (95) urged that this concept of age friendliness represents a critical reflection on the traditional single-group-oriented approach in community planning, marking a shift from “compensatory differentiation” to “systemic inclusion.” Therefore, it should be acknowledged that robust and amicable intergenerational relationships are a crucial component of AFCs and an inclusive, livable society (90). The appeal of being age-friendly lies in its holistic approach, which encompasses not merely being “friendly to older adults,” but also fostering friendliness toward individuals of all ages (91), as exemplified by OMGCs. The core essence of implementing AFCs at the community level resides in ensuring inclusivity and accessibility for all age demographics.

Urgent questions of initiative sustainability have taken hold in the academic literature and among advocates implementing age-friendly changes (54, 78). Internationally, numerous studies have provided multi-dimensional insights into enhancing the sustainable development capacity of urban spaces (92, 94). Some studies have proposed that age-friendly sustainability may be conceptualized as an implementation gap between early development stages and long-term viability (18). This conceptualization provided an analytical anchor point for this study to compare the sustainability of GRCs and OMGCs in China. The findings indicated that OMGCs exhibited a stronger capacity for sustainable development compared with GRCs. This was because the vision embedded in the closed logic of GRCs proved unattainable in long-term operational practice. In contrast, OMGCs adopted an open logic from the outset, demonstrating greater adaptability and the ability to achieve long-term sustainable development. Therefore, this study argued that “openness” was a crucial element for the sustainable development of AFCs. Its core lied in transcending the physical boundaries of the community, welcoming a broader range of urban residents, and giving urban residents the opportunities to enjoy the benefits of the city’s economic growth (81). Simultaneously, adopting a market-oriented approach made communities meet a wider spectrum of needs and accommodate more intricate changes.

This research had certain limitations. First, the age-friendly analysis framework developed in this study, which encompassed dimensions such as policies, facilities, services, intergenerational relationships, and sustainability, served as a valuable tool for understanding age-friendly initiatives in Asian countries. Nevertheless, this framework possessed significant potential for further expansion and refinement. Second, this research primarily examined age-friendly practices within the Chinese context from a top-down perspective, which did not fully capture the comprehensive complexity of age-friendly efforts in China. Lopes et al. (93) emphasized that the inclusion of stakeholders’ comments and suggestions in development of sustainable and inclusive city contributes to improving the quality of decision-making, to gaining greater acceptance of policies, and to widening the understanding of problems. This article did not adequately consider the role of local residents in shaping or developing AFCs, as well as the increasingly pronounced demands and advocacy from local residents in communities, which is a deficiency of the article. Further research is needed to incorporate these perspectives in the future. Third, this research has elucidated the dynamics of these two community types at their current stage of development. Yet, it requires further theoretical elaboration and ongoing monitoring to construct a more systematic knowledge framework. Despite these limitations, the merit of this research was focusing on age-friendly initiatives in developing countries and Asian nations, thereby enhancing the understanding of age-friendly initiatives in diverse contexts. Additionally, it documented Chinese explorations into AFCs and identified effective measures to foster age-friendliness at the community level. As an innovative study, it offers meaningful insights for countries that are either preparing or have recently commenced exploring the development of age-friendly initiatives. Consequently, this remains a valuable contribution to the field.

## 6 Conclusion

This research examined the progress of age-friendly initiatives in China, illustrating how Gated Retirement Communities (GRCs) and Open Multi-Generational Communities (OMGCs) contributed to age-friendliness within the Chinese context, while also addressing the remaining challenges. It identified key priorities for fostering age-friendliness at the community level in China. The study revealed that GRCs were susceptible to issues including spatial injustice, resource waste and high facility idleness rates, service commitment breaches, age discrimination and intergenerational exclusion, and unsustainability, which starkly diverged from age-friendly objectives. By contrast, OMGCs exhibited a higher degree of age-friendliness and greater vitality. Through comparative analysis of the two models, several key priorities for advancing age-friendliness at the community level have been identified: implementing renewal strategies for ordinary existing established mixed-age communities to transform them into more inclusive and supportive OMGCs can extend age-friendly benefits to broader populations; tailoring age-friendly facility configurations to local needs while prioritizing service commitment fulfillment represents a key measure to promote age-friendliness; as intergenerational interaction is a vital dimension of age-friendliness, the spaces that are welcoming to all age rather than exclusively targeting older adults should be created; and integrating with broader urban development is essential for sustaining long-term viability of AFCs. These findings can inform other countries in developing age-friendly policies and establishing AFCs. In the context of an increasingly aging global population, this research holds significant implications for enhancing the well-being of older adults, promoting intergenerational harmony, and advancing social cohesion. Future research should focus on reporting and evaluating the progress of age-friendly initiatives in China and other non-Western and developing settings from a more micro-level perspective, such as the resident participation within AFCs, and through a lens of precise statistical data. These contents are crucial for advancing the establishment of Global Age-friendly City and Community networks.

## Data Availability

The original contributions presented in the study are included in the article/supplementary material, further inquiries can be directed to the corresponding author.
